# Speaking up for the safety of the children following frozen embryo transfer

**DOI:** 10.1093/hropen/hoae058

**Published:** 2024-09-30

**Authors:** Anja Pinborg, Christophe Blockeel, Giovanni Coticchio, Juan Garcia-Velasco, Pietro Santulli, Alison Campbell

**Affiliations:** Fertility Clinic, Department of Gynecology, Fertility and Obstetrics, Rigshospitalet, Copenhagen University Hospital, Copenhagen, Denmark; Department of Clinical Medicine, Copenhagen University, Copenhagen, Denmark; Brussels IVF, Centre for Reproductive Medicine, Universitair Ziekenhuis Brussel, Brussels, Belgium; IVIRMA Global Research Alliance, IVIRMA ITALIA, Rome, Italy; IVIRMA Global Research Alliance, IVIRMA, Madrid, Spain; Department of Medical Specialties and Public Health, Rey Juan Carlos University, Madrid, Spain; Faculté de Santé, Faculté de Médecine Paris Centre, Université de Paris Cité, Paris, France; Department of Gynecology Obstetrics II and Reproductive Medicine, Assistance Publique-Hôpitaux de Paris (AP-HP), Centre Hospitalier Universitaire (CHU) Cochin, INSERM U1016, Paris, France; Care Fertility, Nottingham, UK; University of Kent, Canterbury, UK

Since the first IVF child was born in 1978, more than 10 million children have been born through various assisted reproductive technologies. Although the first child was born from a frozen embryo in 1983, cryopreservation began to have a more significant impact in the early 2000s, thanks to vitrification techniques and improvements that enable the extension of embryo culture up to day 5–7 of development. Together, these two advancements have led to significantly improved embryo selection and higher post-cryopreservation survival rates—up to 99% (Rienzi *et al.*, 2017). Consequently, pregnancy and live birth rates after cryopreservation are now as high as those after fresh embryo transfer, and the number of frozen cycles has exceeded fresh embryo transfers in many countries.

Furthermore, embryo cryopreservation has facilitated single embryo transfers, as surplus embryos can be efficiently stored for later use. This has aided the reduction of multiple pregnancies to a minimum in countries where single embryo transfer has been implemented as the standard of care, which mirrors the background population rate of 2–3% (although today, the average multiple birth rate after ART in Europe is 12.2%) (European IVF Monitoring Consortium (EIM) for the European Society of Human Reproduction and Embryology (ESHRE) *et al.*, 2023). This contrasts with multiple birth rates of up to 30% that were seen at the beginning of the IVF era. The single embryo transfer policy has resulted in a significant reduction in preterm birth rates within the IVF population and, importantly, the rate of cerebral palsy has decreased from an incidence that was 2–3 times higher than the general population to comparable levels (Spangmose *et al.*, 2021).

A positive consequence of vitrification, along with refined ART treatment protocols, is that ovarian hyperstimulation syndrome (OHSS) can be eradicated by applying ovarian stimulation using a GnRH antagonist (short protocol) with a GnRH agonist to trigger ovulation in combination with a ‘freeze-all’ strategy (Blockeel *et al.*, 2019). Postponing embryo transfer to a cycle without ovarian stimulation results in a dramatic reduction in the prevalence of OHSS (Devroey *et al.*, 2011), which has been a game-changer for people undergoing ART treatment and has greatly improved safety profiles.

Additionally, reproductive medicine has greatly benefited from preimplantation genetic testing for aneuploidies (PGT-A), which is routinely performed in freeze-all cycles without compromising embryo survival rates. While the generic use of PGT-A remains controversial, its effectiveness has been largely accepted for specific patient groups, such as women with recurrent pregnancy loss and recurrent implantation failure. With this combined PGT-A and freeze-all approach, miscarriage rates and time to live birth may be reduced. However, further studies are required to ensure that cumulative live birth rates are not affected, and to more strictly define other indications and limitations of this treatment strategy (ESHRE Add-ons working group *et al.*, 2023; Human Fertilisation and Embryology Authority, 2024).

Unsurprisingly, the enthusiasm for new procedures associated with improved cryopreservation techniques has been overwhelming. Countless conferences have seen debates on ‘freeze-all for all’ and PGT-A and, in many settings, these two strategies in combination are now advocated as the ‘gold standard’. Nevertheless, nothing comes without a price—and cryopreservation of embryos is no exception. Recently, large cohort and international register studies have shown that the risks of preeclampsia and large-for-gestational age babies are significantly higher in pregnancies after freezing and thawing of embryos, compared with fresh embryos, in maternal factor-controlled sibling embryo studies (Petersen *et al.*, 2023). Furthermore, frozen embryo transfer (FET) protocols with hormone replacement therapy (sequential estradiol and progesterone) named artificial cycle FET (AC-FET), which prepares the endometrium without ovulation—thus, in the absence of a corpus luteum—further increases this risk of preeclampsia (Zaat *et al.*, 2023). AC-FET has been used extensively owing to its convenience for laboratory and clinic scheduling of embryo thawing and transfer, as opposed to natural FET cycles where scheduling is led by the detection of ovulation with repeated LH measurements or ultrasound monitoring of the maturing follicle (Løssl *et al.*, 2023).

Recently, two large cohort studies based on national registry data in France and the Nordic countries (including 260 236 and 171 774 ART children, respectively) have shown a higher risk of cancer, particularly leukemia, in children born after FET (Sargisian *et al.*, 2022; Rios *et al.*, 2024). The incidence rate was 30.1 for FET and 18.8 for fresh embryo transfer per 100 000 children in comparison to 16.7 per 100 000 children in naturally conceived children within the Nordic population. This gives an adjusted risk that is 1.65-fold higher for FET compared with the background Nordic population. This is very similar to the 1.61-fold increased risk that was reported within the French population. However, these risks are based on small absolute numbers and should be interpreted with caution. While cryopreservation of embryos is an essential element of ART treatment, the findings from these large studies suggest that a mindful and cautionary approach toward freeze-all practices should be taken, such that they are only used where clearly indicated.

Continuous surveillance of the short- and long-term consequences of ART treatments should be prioritized by healthcare authorities, and should be based on the collection and analysis of data collected by the HFEA (Human Fertilisation and Embryology Authority) in the UK, the BELRAP (Belgian Register for Assisted Procreation) in Belgium, the FIVNAT (Fécondation In Vitro National) in France, and the Swedish and Danish quality registries on ART (now being implemented in Norway). This will enable us to survey and assess the consequences of ART and develop strategies to increase the safety and effectiveness of treatments thereafter (Pinborg *et al.*, 2023).

Reproductive medicine is a relatively new specialty in the medical field, and people suffering from infertility will go to great lengths to fulfil their dream of having a child. Therefore, we have a responsibility to survey the treatments and new technologies available and only use them by indication. Recommendations and guidelines have been developed by authorities in the UK (HFEA) and on behalf of ESHRE to try to curb the increased use of non-evidence-based treatments: the so-called ‘add-ons’ (ESHRE Add-ons working group *et al.*, 2023; Human Fertilisation and Embryology Authority, 2024). The financial factor is significant and, as ART is mostly performed without governmental reimbursement, there is pressure from many stakeholders including patients who want to have a child, their families, health authorities, government, and fertility clinic financial investors—to whom ART is a business. This may influence decisions on treatment strategies.

In conclusion, considering the risk profiles associated with cryopreservation of embryos highlighted by large cohort studies, cryopreservation should be used for storing surplus embryos, in cases of high risk of OHSS, or if PGT-A is indicated, but a ‘freeze-all’ approach should not be universally applied during ART treatment.

## Authors’ roles

All authors contributed to the conception of the paper, the drafting, critical reading, and final approval of the submitted version of the manuscript.

## References

[hoae058-B1] Blockeel C , Campbell A, Coticchio G, Esler J, Garcia-Velasco JA, Santulli P, Pinborg A. Should we still perform fresh embryo transfers in ART? Hum Reprod 2019;34:2319–2329. 31803911 10.1093/humrep/dez233

[hoae058-B2] Devroey P , Polyzos NP, Blockeel C. An OHSS-Free Clinic by segmentation of IVF treatment. Hum Reprod 2011;26:2593–2597. 21828116 10.1093/humrep/der251

[hoae058-B3] ESHRE Add-ons working group; Lundin K, Bentzen JG, Bozdag G, Ebner T, Harper J, Le Clef N, Moffett A, Norcross S, Polyzos NP, Rautakallio-Hokkanen S, et al Good practice recommendations on add-ons in reproductive medicine. Hum Reprod 2023;38:2062–2104. 37747409 10.1093/humrep/dead184 PMC10628516

[hoae058-B4] European IVF Monitoring Consortium (EIM) for the European Society of Human Reproduction and Embryology (ESHRE); Smeenk J, Wyns C, De Geyter C, Kupka M, Bergh C, Cuevas Saiz I, De Neubourg D, Rezabek K, Tandler-Schneider A, Rugescu I, et al ART in Europe, 2019: results generated from European registries by ESHRE. European. Hum Reprod 2023;38:2321–2338. 37847771 10.1093/humrep/dead197 PMC10694409

[hoae058-B5] Human Fertilisation and Embryology Authority. Pre-Implantation Genetic Testing for Aneuploidy (PGT-A). 2024. https://www.hfea.gov.uk/treatments/treatment-add-ons/pre-implantation-genetic-testing-for-aneuploidy-pgt-a/ (01 July 2024, date last accessed).

[hoae058-B6] Løssl K , Spangmose AL, Asserhøj LL, Dam TV, Pinborg A. The future of frozen-thawed embryo transfer in hormone replacement therapy cycles. Curr Opin Obstet Gynecol 2023;35:200–209. 37185352 10.1097/GCO.0000000000000867

[hoae058-B7] Petersen HS , Westvik-Johari K, Spangmose AL, Pinborg A, Romundstad LB, Bergh C, Åsvold BO, Gissler M, Tiitinen A, Wennerholm UB et al Risk of hypertensive disorders in pregnancy after fresh and frozen embryo transfer in assisted reproduction: a population-based cohort study with within-sibship analysis. Hypertension 2023;80:e6–e16. 36154568 10.1161/HYPERTENSIONAHA.122.19689

[hoae058-B8] Pinborg A , Blockeel C, Campbell A, Coticchio G, Garcia-Velasco JA, Santulli P, De Geyter C, Wyns C. The time has come for harmonized international ART registration. Reprod Biomed Online 2023;46:881–885. 37024399 10.1016/j.rbmo.2023.02.015

[hoae058-B9] Rienzi L , Gracia C, Maggiulli R, LaBarbera AR, Kaser DJ, Ubaldi FM, Vanderpoel S, Racowsky C. Oocyte, embryo and blastocyst cryopreservation in ART: systematic review and meta-analysis comparing slow-freezing versus vitrification to produce evidence for the development of global guidance. Hum Reprod Update 2017;23:139–155. 27827818 10.1093/humupd/dmw038 PMC5850862

[hoae058-B10] Rios P , Herlemont P, Fauque P, Lacour B, Jouannet P, Weill A, Zureik M, Clavel J, Dray-Spira R. Medically assisted reproduction and risk of cancer among offspring. JAMA Netw Open 2024;7:e249429. 38696167 10.1001/jamanetworkopen.2024.9429 PMC11066701

[hoae058-B11] Sargisian N , Lannering B, Petzold M, Opdahl S, Gissler M, Pinborg A, Henningsen AA, Tiitinen A, Romundstad LB, Spangmose AL et al Cancer in children born after frozen-thawed embryo transfer: a cohort study. PLoS Med 2022;19:e1004078. 36048761 10.1371/journal.pmed.1004078 PMC9436139

[hoae058-B12] Spangmose AL , Christensen LH, Henningsen AA, Forman J, Opdahl S, Romundstad LB, Himmelmann K, Bergh C, Wennerholm UB, Tiitinen A et al Cerebral palsy in ART children has declined substantially over time: a Nordic study from the CoNARTaS group. Hum Reprod 2021;36:2358–2370. 34051081 10.1093/humrep/deab122

[hoae058-B13] Zaat TR , Kostova EB, Korsen P, Showell MG, Mol F, van Wely M. Obstetric and neonatal outcomes after natural versus artificial cycle frozen embryo transfer and the role of luteal phase support: a systematic review and meta-analysis. Hum Reprod Update 2023;29:634–654. 37172270 10.1093/humupd/dmad011 PMC10477943

